# Childhood Tuberculosis and Isoniazid Preventive Therapy Among Household Contacts of Sputum Positive Pulmonary Tuberculosis Patients: A Descriptive Observational Study

**DOI:** 10.7759/cureus.26643

**Published:** 2022-07-07

**Authors:** Namita Shanbag, Mohammad Haseeb, Madhuri Engade, Mohd Saeed Siddiqui, Khaled M Badaam, Madhurasree Nelanuthala

**Affiliations:** 1 Pediatrics and Child Health, Dr. Ajayans Hospital, Navi Mumbai, IND; 2 Pediatrics, Mahatma Gandhi Mission (MGM) Medical College and Hospital, Aurangabad, IND; 3 Physiology, Government Medical College (GMC) Aurangabad, Aurangabad, IND

**Keywords:** rntcp, contact screening, childhood tuberculosis, contact tracing, pulmonary tuberculosis

## Abstract

Background

The prevalence of tuberculosis (TB) disease among household contacts of adult TB patients is very high. Contact screening and isoniazid preventive therapy (IPT) are recommended for household contacts, but their uptake by families and implementation by the national TB program are poor. This study was performed to estimate the yield of tuberculosis disease, risk factors associated with disease development, and coverage of IPT in household contacts of sputum-positive pulmonary tuberculosis patients in the Aurangabad district of Maharashtra.

Methods

A cross-sectional study was conducted at MGM Medical College Hospital Aurangabad. Sputum-positive adult TB patients were enrolled in the study. Their household contacts were screened for TB disease, and the status of IPT in eligible contacts was studied. Serial screening and follow-up of these contacts were performed for one year.

Results

A total of 82 contacts of 55 index cases were studied. At the one-year follow-up, 15 (18%) patients developed TB disease. Twelve had pulmonary TB, and three had extrapulmonary TB. The mean age of diseased contacts was 5.5 years. The disease was more common in contacts under six years of age. Sixty-four percent of eligible contacts received IPT. There was a statistically significant association between disease development and noncompliance with IPT (p-value 0.005913).

Conclusions

The yield of tuberculosis disease is high in children contacts with sputum-positive pulmonary TB cases. IPT implementation is inadequate in child contact.

## Introduction

Tuberculosis (TB) causes significant morbidity and mortality in children in many TB endemic countries. At least 550,000 children become ill with TB each year globally [1]. The incidence of TB disease among household contacts is very high, reported as >1,000 cases/100,000 population [2]. The likelihood of infection depends on the patient's infectiousness, duration and proximity of the contact with the index case, and susceptibility of the contact [3-6]. The disease onset may occur early, within six weeks of exposure or many years later [7]. TB contacts have a significantly increased risk of all-cause mortality compared to children living in non-TB households in the same community.

India's Revised National Tuberculosis Control Program (RNTCP) recommends screening all household contacts of smear-positive pulmonary TB (PTB) cases for TB disease and six months of isoniazid preventive therapy (IPT) for asymptomatic children aged <6 years [8]. The recommendation can significantly reduce the burden of TB in children. However, the uptake by the families and implementation of the national tuberculosis programs (NTPs) is poor. An Indian study reported that only 14% of children younger than fourteen years living in the same house as adults with pulmonary TB were screened for TB. Another study from India reported that 19% of child contacts younger than six years were initiated on IPT [9,10].

The present study assesses the burden of TB disease and IPT coverage in pediatric household contacts of newly diagnosed adult TB patients registered with the RNTCP unit of Mahatma Gandhi Mission's Medical College and Hospital, Aurangabad.

## Materials and methods

Study design

The present observational & cross-sectional study was conducted at the Mahatma Gandhi Mission's Medical College & Hospital (MGM MCH), a tertiary care center in Aurangabad (Maharashtra, India), between October 2017 to October 2019. The primary outcome was the proportion of child TB contacts screened for TB, prescribed IPT, and the proportion of child contacts diagnosed with active TB. Compliance with IPT and the status of diseased children were secondary outcomes. 

Ethical considerations

The study was approved by the Mahatma Gandhi mission's ethics committee for research on human subjects (vide letter number - MGM-ECRHS 2017/50). Written and informed consent was obtained from the parents of study participants before enrollment.

Inclusion Criteria

a) All children under the age of five years who had lived in the same home as the index case for at least three months.

b) Children of newly diagnosed TB patients regularly visit MGM MCH Aurangabad for anti-TB treatment during the study period.

Exclusion Criteria

Children who did not complete the baseline assessment or who failed to show up for at least two follow-up visits were excluded.

Data collection

Eligible household contacts were enrolled by simple random sampling from the RNTCP TB unit of MGM Medical College Hospital Aurangabad. The index or source case is a newly diagnosed adult sputum-positive pulmonary TB patient. Contact cases are household contacts of these index cases in the age group of six months to fifteen years. These household contacts were screened at the time of enrollment, and their follow-up was performed over one year. At the time of the first visit, detailed history was taken, and a physical examination of all household child contacts was done. Symptomatic cases were further analyzed by TST (tuberculin skin test), chest X-ray, and sputum smear examination by microscopy and cartridge-based nucleic acid amplification test (CBNAAT).

According to the results of the investigations, child contacts were labeled as diseased or not diseased. We further categorized the diseased contacts as clinically diagnosed or microbiologically confirmed. These diseased contacts were registered under RNTCP for treatment. The IPT status of asymptomatic child contacts <6 years of age was noted. If they were not on IPT, we advised them of an INH (isoniazid) prophylaxis therapy of 5 mg/kg/day for six months. All enrolled child contacts were asked for follow-up after six months and 12 months of enrollment and whenever they were symptomatic. Cases not coming for follow-up were considered dropouts. On follow-up visits, we assessed asymptomatic child contacts under six years for adherence to IPT (INH prophylaxis therapy) and the diseased child contacts for compliance to anti-tubercular drugs and their side effects. The same protocol was repeated at follow-up visits. At the end of the study, we analyzed risk factors for developing the disease in household child contacts of sputum-positive adult pulmonary TB patients.

Case definitions

An index case or a source case is defined as the first sputum smear-positive pulmonary TB case identified in the household. We defined child contacts as children living for at least three months in the same household as the index case during their disease until the end of treatment. Symptomatic child contact includes a child having contact with the index or a source case with a history of symptoms such as persistent cough, fever, or weight loss. Persistent cough was an unremitting cough for >21 days that did not improve with regular medications. Fever was defined as a body temperature of >38 degrees Celsius or 100.4 Fahrenheit recorded over several hospital visits for 14 days when other common causes, such as malaria and pneumonia, were ruled out. Weight loss or failure to thrive was noticed on a child's plotted growth chart or decline in weight from previously recorded weights and not just as stated by parents.

Diseased child contact was further classified as microbiologically confirmed or clinically diagnosed as follows: 1. Clinically diagnosed- At least one well-defined symptom suggestive of TB with positive radiological evidence in the form of hilar lymphadenopathy or intrathoracic finding of TB on CXR and USG representative of necrotic matted lymph nodes in case of extrapulmonary TB with associated positive clinical response to anti-tuberculosis treatment. 2. Microbiologically confirmed- sputum smear-positive, culture, or CBNAAT confirmation of mycobacterium tuberculosis.

Statistical analysis

We compiled all the data in an Excel spreadsheet (Microsoft Corporation, Redmond, WA) and a master chart was prepared. We used BM SPSS Statistics for Windows, Version 20.0 (IBM Corp., Armonk, NY) for data analysis. Qualitative data were expressed in frequency and percentage, and quantitative data were expressed in terms of mean ± SD. The chi-square test was used to assess associations.

## Results

During the study period (October 2017 to October 2019), 133 sputum-positive adult patients (index cases) were registered with the RNTCP unit at MGM Hospital Aurangabad. Out of these 133 index cases, 60 cases were enrolled in the study by a simple random sampling method, and five enrolled cases did not appear for follow-up visits, so 55 index (source) cases were included in the study. These 55 sputum positive index cases had 82 household child contacts (children up to 15 years of age), they were screened and studied.

The mean age of the index case was 33 years, of which 29 were males and 26 were females (Table 1).

**Table 1:** Demographic details of Index cases Values given in parenthesis are percentages, n-number.

Characteristics	Index cases, n=55
Age (Mean±SD)	33.12 ± 11.51 years
21-30	29 (52.7)
31-40	17 (30.9)
41-50	06 (10.9)
>50	03 (5.4)
Gender	
male	29 (52.72)
female	26 (47.27)

The mean age of the 82 household contacts was 6.7 years. Forty-five (55%) contacts were less than six years of age, with a mean age of 2.7 years, and 37 (45%) contacts were more than six years of age, with a mean age of 8.9 years. The male-to-female ratio was 1.8:1 (49 male and 33 female contacts). Forty-five (55%) were eligible for IPT prophylaxis, of them 29 (64.44%) were on IPT. Fifteen (18.29%) developed disease at the end of the study, twelve of them had pulmonary TB and three had extra pulmonary TB (Table 2).

**Table 2:** Demographical and clinical details of household contacts Values given in parenthesis are percentage, n - number. IPT - isoniazid prophylactic therapy, TB - tuberculosis

Characteristics	Household Contacts, n=82
Age (mean)	6.7
<6 years	45 (55)
>6 years	37 (45)
Sex	
Male	49 (59.75)
Female	33 (40.24)
IPT	
Advised	29 (64.44)
Not Advised	16 (35.55)
Duration of Contact	
< 6 months	60 (73.17)
> 6 months	22 (26.82)
Outcome	
Developed TB	15 (18.29)
Healthy household contacts	67 (81.7)
Pulmonary TB	12 (80)
Extra Pulmonary TB	3 (20)
Relation of contact with index case	
Father	28 (34.1)
Mother	27 (32.9)
Grandparents	11 (13.4)
Others	16 (19.5)
Nutritional status	
Malnourished	31 (37.8)
Normal nutritional status	51 (62.1)

The mean age of these diseased child contacts was 5.5 years. A total of 62.5% of them were under the age of six years. A chi-square test of independence showed that there was no significant association between age and disease development (p=0.31). The same test was performed to examine the relation between gender and the development of TB among household contacts. The relation between these variables was significant (p=0.000001); thus, male contacts were more likely to have the disease than female contacts. Similarly, a significant association was noted between contact duration and disease development (p=0.010376). In studying the relationship between index case and diseased contact, we found that the index case was mother in seven contacts, grandparent in four and father in three and another family member like an uncle, aunt in the remaining five diseased contacts. In nondiseased contacts, the index case was their father (25 cases), followed by their mother (20 cases), a grandparent (7) and others (11). The association between the development of disease and the relationship of contact was not statistically significant (p=0.238885).

Forty-five child contacts were under the age of six years, and they were eligible for IPT, but out of these 45 contacts, 29 (64%) received IPT. Of the 15 child contacts who were diseased, 10 were eligible for IPT; however, they did not complete the IPT course. We found a significant association between nonintake of IPT and disease development in household contacts (p-value 0.00205). After completion of six-month AKT as per RNTCP protocol, all diseased contacts were declared cured (Table 3).

**Table 3:** Factors associated with incident tuberculosis cases among household contacts of index cases Values given in parenthesis are percentage, n-number. IPT-isoniazid prophylactic therapy, TB- tuberculosis

parameter	Healthy household contacts, n=67	Household contacts with TB, n = 15	P value
Age (mean)-6.7			0.310085
Age < 6 years	35 (52.2)	10 (62.5)	
Age > 6 years	32 (47.7)	5 (38.5)	
Gender			0.00001
Male	10 (14.9%)	11 (73.3)	
Female	57 (85.07)	4 (26.6)	
IPT status			0.00205
On IPT	24 (35.8)	5 (33.3)	
Without IPT	6 (8.95)	10 (66.6)	
Contact duration			0.010376
<6 months	53 (79.1)	7 (46.6)	
>6 months	14 (20.8)	8 (53.3)	
Nutritional status			0.846204
Malnourished	25	6	31
Normal nutritional status	42	9	51
Relation of contact with index case			0.238885
Father	25	3	
Mother	20	7	
Grandparents	7	4	
Others	11	5	

## Discussion

In this cross-sectional study, we analyzed household contacts of newly diagnosed adult TB patients registered with the RNTCP unit MGM MCH Aurangabad for the burden of TB disease, IPT coverage, and risk factors related to the disease's development. In this study, we found that 18% of child contacts of index TB cases either had prevalent TB upon screening or developed the disease shortly afterward. In Bayer's study performed in South Africa, the yield of TB in household child contacts was 27% [11]. Various previous studies recorded a low prevalence (1% to 10%) [12-15]. A higher yield of 52% was reported by Tornee et al. in a prospective study in Thailand [16]. The likelihood of infection is related to IPT initiation, the bacillary load of the index case, and the closeness of contact with the index case. The risk of infection is greatest when the index case is the child's caregiver, e.g., mother or grandmother [3,4]. But in the present study, we found no significant association between the closeness of contact and disease development (p = 0.238885). Only sputum-positive pulmonary TB adults as index cases and low IPT coverage (64%) might have contributed to the high incidence of disease in our study population. Many of our index cases were from urban slums, and their living conditions also contributed to the high transmission rate. In our study, we performed a serial screening for TB child contact cases over one year, which also increased the disease yield. There is evidence of a substantial TB incidence within five years of exposure, particularly within the first 12 months. It highlights the potential importance of serial screening for TB in contacts who do not receive treatment for latent infection [17].

In the present study, 11 (73%) of 15 diseased children were diagnosed by the investigating officer, and four were diagnosed through RNTCP. Under normal circumstances, these 11 children would have either remained undiagnosed or were diagnosed at an advanced stage of the disease. Children with TB often present with subtle or nonspecific clinical features, and its diagnosis requires a high degree of clinical suspicion and frequent follow-up visits. Under the RNTCP, the paramedical health worker performs initial screening and home visits. The problems that paramedical health workers encountered primarily included health system-related issues such as the requirement for patients and contacts to attend the health center several times and a lack of staff experience in interpreting diagnostic data. These difficulties have been identified in previous studies [6,10]. In our study, regular evaluation by a pediatrician enhanced the diagnostic efficiency.

We had 82 child contacts in a 1:1.5 ratio for 55 index cases. The mean age of the index case was 35 years, which also explains more children under six years of age in the contact population. The results are similar to a study performed in northern India [18]. We also observed more younger children in the diseased group than in the older group. In the present study, 55% of diseased contacts were under six years of age. The association between contact age and disease development was insignificant (p-value 0.310085).

Twenty-nine (64%) of asymptomatic contacts under six years received IPT in the present study. Banurekha et al. study noted 59% IPT coverage in eligible household contacts [14]. This coverage is lower than the findings (74% and 84%) of previous studies performed in northern and southern India [15-19], but IPT coverage in our study population was better than the variable findings (6%-28%) of other previous studies [10,20-22]. In general, factors associated with poor IPT coverage were lack of screening, poor adherence to prescribed IPT, inadequate patient and family counseling, poor acceptability of IPT by family due to lack of knowledge about the protective efficacy of IPT, in some cases, non-availability of IPT and inadequate monitoring of the implementation of this guideline. We did not analyze factors responsible for poor IPT coverage in individual cases in this study. IPT reduces the risk of TB disease by approximately 60% among infected contacts of all ages [23]. Our study shows suboptimal administration of contact screening and IPT in the study population. A policy is universally accepted but seldom implemented. Contact screening helps in the early diagnosis and initiation of treatment, and IPT is an effective way of preventing TB in child contacts. We need to emphasize the role of IPT in preventing TB in children.

The study has some limitations, like purposively selected health facilities, so the findings might not be generalizable. Also, the unavailability of detailed data analyzing factors responsible for inadequate contact screening and IPT initiation can be considered a limitation. However, our results provided program-level evidence about the actual implementation of contact screening and IPT.

## Conclusions

Childhood tuberculosis is a growing concern in the fight against TB. The yield of tuberculosis disease is high in children in contact with sputum-positive pulmonary TB cases. Contact screening is an effective method for the early diagnosis of TB among child contacts. Despite World Health Organization and RNTCP guidelines, pediatric contact screening and IPT implementation under routine programmatic conditions are suboptimal. Failure to screen and routinely provide IPT for childhood contacts results in multiple missed opportunities for diagnosing and preventing pediatric TB cases. Significant gaps in non-initiation may be corrected by staff training, enhanced supervision, and monitoring. Further studies are needed to understand better factors contributing to suboptimal contact screening and IPT initiation in child contacts.
